# The role of Rwanda Field Epidemiology and Laboratory Training Program graduates and residents in response to the first Marburg Virus Disease outbreak in Rwanda

**DOI:** 10.1371/journal.pgph.0004452

**Published:** 2025-09-12

**Authors:** Edouard Ruseesa, Aphrodis Hagabimana, Fredrick Ntirenganya, Peace Kinani, Ruton Hinda, Emmanuel Nshimiyimana, Claude Niyoyita, Angela Umutoni, Caroline Stamatakis, Hugues Valois Mucunguzi, Edson Rwagasore

**Affiliations:** 1 Public Health Surveillance and Emergency Preparedness and Response Division, Rwanda Biomedical Centre, Kigali, Rwanda; 2 Rwanda Field Epidemiology and Laboratory Training Program, Department of Epidemiology and Biostatistics, University of Rwanda, Kigali, Rwanda; 3 African Field Epidemiology Network, Kigali, Rwanda; 4 Centers for Disease Control and Prevention, Division of Global HIV and TB, Kigali, Rwanda; University of St. Andrews, UNITED KINGDOM OF GREAT BRITAIN AND NORTHERN IRELAND

## Abstract

Rwanda Field epidemiology and laboratory training programs (RFELTP) build capacity to improve disease surveillance, respond to public health emergencies, and inform evidence-based decision-making to mitigate the impact of epidemics and other public health events. On September 27, 2024, Rwanda declared its first Marburg virus disease (MVD) outbreak. MVD disease is a severe haemorrhagic fever with a fatality rate ranging from 24% to 90%. We describe the role played by the RFELTP in the outbreak response, and identify key strengths and challenges of the program related to the response. In November 2024, we provided a self-administered questionnaire delivered online to all residents and graduates of RFELTP. The questionnaire included sections on sociodemographic, experience in outbreak response, and MVD outbreak response experiences. Descriptive statistical analysis was performed, and the data were analysed using Stata version 16. Among the 523 total participants, 209 responded (40.0%). About a third of the participants (34.8%) supported MVD response activities. Residents and graduates supported the response in all 30 districts of Rwanda, mainly at the national and district levels. Most supported epidemiology and surveillance 41.2%, data management 19.2%, infection prevention control 14.3%, and leadership and coordination pillars 10.4%. Nearly all 96.7% felt adequately prepared by their RFELTP to handle outbreak responses. The RFELTP graduates and residents proved to be a critical resource in the MVD outbreak response, enhancing the country’s capacity to respond to public health emergencies. Continued investment in this training program is essential for enhancing global health security and strengthening capabilities to respond to public health emergencies.

## Introduction

The Rwanda Field Epidemiology and Laboratory Training Program (RFELTP) is a competency-based training program aimed at strengthening field epidemiology capacity at all levels of the health system to prevent, detect, investigate and respond to public health priority issues [1]. RFELTP is led by the Government of Rwanda, with assistance from the University of Rwanda’s School of Public Health, African Field Epidemiology Network, as well as technical support and funding from the U.S. Centers for Disease Control and Prevention. The program consists of three tiers: Frontline, Intermediate, and Advanced. Established in 2009, the Advanced Tier consists of both didactic components and field placements that facilitate competency development in outbreak investigation, surveillance systems, data analysis, protocol-based studies, and scientific communication. To date, the program has trained 91 field epidemiologists who are primarily deployed at the national and district levels to provide public health leadership and technical expertise. The Intermediate Tier was launched in 2022 as a nine-month training program that has a similar curriculum structure as the Advanced training, but mainly targets district-level health officials. As of December 2024, 33 Intermediate RFELTP residents have been trained. The Frontline Tier was introduced in 2020 as a three-month program, largely in response to the COVID-19 pandemic. It focuses on sub-national levels of the health sector and consists of three on-site training workshops, and has two field placements during which participants complete field projects in their primary work sites throughout Rwanda. Through the Frontline Tier, 399 participants have been trained, including clinical directors from hospitals, integrated disease surveillance and response officers, monitoring and evaluation officers, health promotion officers, animal resources officers and others. In total, the RFELTP has trained 523 professionals (including graduates and current residents) in field epidemiology across the three tiers (Frontline: 399, Intermediate: 33, Advanced: 91). Graduates serve at various health administrative levels in Rwanda and make up the country’s primary roster of surge staff available to respond to urgent public health situations. They have successfully completed either the advanced, intermediate or frontline training. Residents are individuals still in the RFELTP advanced, intermediate, or frontline training cohorts.

MVD is a severe and often fatal haemorrhagic fever caused by infection with the Marburg virus [2,3]. MVD, which belongs to the same family (*Filoviridae*) as Ebola virus, has a case-fatality rate that ranges from 24% to 90% [4–6]. Egyptian fruit bats (*Rousettus aegyptiacus*) are the reservoirs of MV [7–9]. Transmission of the virus occurs through either direct or indirect contact between fruit bats and humans and through human-to-human transmission with an estimated 2–21 days incubation period [10].

On 27 September 2024, Rwanda’s Ministry of Health (MoH) declared the nation’s first-ever MVD outbreak. In total, 66 cases were confirmed, including 15 deaths, resulting in a case-fatality rate of 22.7% [11]. All confirmed cases were detected from the three districts in the capital, Kigali City. Kigali is characterized by its dense population and significant influx of people from various districts and foreign countries [12], creating conditions that potentially increase the risk of disease transmission. Health workers from two health facilities in Kigali, King Faisal Hospital and the University Teaching Hospital of Kigali, accounted for the majority of confirmed cases. Confirmed cases who were not healthcare workers were also linked to these health facilities [13].

Effectively managing an MVD outbreak requires a comprehensive strategy encompassing several key measures. These include early case identification, patient isolation and treatment, disease surveillance, systematic contact tracing and quarantine, infection prevention and control, robust laboratory diagnostic capabilities, safe and dignified burials, and effective risk communication and community engagement [14]. The effectiveness of these interventions depends on a well-trained public health workforce [15]. The World Health Organisation (WHO) also recognizes a strong public health workforce as critical to compliance with the 2005 International Health Regulations [16].

We aimed to describe the role of RFELTP graduates and residents in response to the MVD outbreak in Rwanda from September to mid-November 2024, and identify key strengths and challenges of the program related to the response.

## Methods

### Ethics statement

The information reported in this article represents data collected during the MVD outbreak response. An outbreak investigation is regarded as an emergency activity and was endorsed by MOH. Permission to collect and publish this information was sought and granted by the Research, Innovation and Data Science (RIDS) Division in Rwanda Biomedical Centre (RBC). In accordance with the Declaration of Helsinki, participation in the survey was voluntary, and informed consent was obtained from all participants prior to completing the survey. At the start of the online questionnaire, participants were presented with an informed consent and the participant checked the “I agree” box to indicate consent before proceeding to the questionnaire. To maintain the confidentiality of respondents, strict measures were implemented to safeguard participant data. During the data collection phase, all personal identifying information was anonymized.

We described the roles of RFELTP graduates and residents in the MVD response to illustrate their engagement and extent of their support during this emergency response. A complete list of all 523 graduates and residents from the Advanced, Intermediate, and Frontline RFELTP tiers was obtained from the University of Rwanda and Rwanda Biomedical Centre (RBC), which oversees the RFELTP program. The RFELTP program administrators, who also served in leadership roles in MVD outbreak response, provided qualitative data on the timeline of response and mobilization efforts to engage the RFELTP graduates and residents in the response.

From 1^st^ to 30^th^ November 2024, we conducted an online survey using a questionnaire designed through the Kobo Toolbox platform [16]. The questionnaire was first piloted by administering it to five people who are among the study participants to assess its clarity, relevance, and ease of understanding before final distribution. All 523 graduates and residents were contacted through phone calls and emails to participate in the online survey. The research team provided guidance and support by phone to answer questions participants raised, to ensure complete and accurate responses.

The survey questionnaire consisted of two main parts. The first section collected demographic data (age, sex, RFELTP training level, training cohort, professional background, current employment, and years of experience in epidemiology and outbreak response). The second section focused on the participants’ specific roles, activities, and challenges encountered during the MVD outbreak response. The survey collected information on involvement across the different response pillars, including leadership and coordination, epidemiological surveillance, laboratory, case management, infection prevention and control, risk communication, vaccination and data management.

Descriptive statistics (frequencies and percentages) were calculated to summarize the participants’ sociodemographic characteristics and their involvement in various aspects of the MVD outbreak response. Data were analysed using Stata version 16 [17] and presented in tables, a map and graphs.

## Results

All of the 523 RFELTP graduates and residents were emailed the survey. Among 209 (40.0%) RFELTP graduates and residents who responded to the survey, a total of 182 (87.1%) were involved in the MVD response that included; 28.6% the advanced tier, 14.8% intermediate tier and 56.6%. Frontline Tier. The majority 55.5% were aged between 35–44 years, 65.9% were male, and 57.3% had more than five years of experience in public health or epidemiology (Table 1).

**Table 1:** Sociodemographic characteristics of the participants, Rwanda, 2024.

Variable	Category	Frequency (n)	Percentage (%)
**Age group**	25-34	44	24.2
	35-44	101	55.5
	45-54	32	17.6
	≥55	5	2.7
**Sex**	Female	62	34.1
	Male	120	65.9
**Highest tier of FELTP**	Advanced	52	28.6
	Intermediate	27	14.8
	Frontline	103	56.6
**Training status**	Graduate	118	64.8
	Residents	64	35.2
**Professional background**	Allied Health Sciences	81	44.5
	Animal Health/Veterinary	8	4.4
	Data Science	28	15.4
	Medical doctor	20	11.0
	Nurse	45	24.7
**Current level of employment**	National	53	29.4
	Province	8	4.4
	District	88	48.9
	Sector	31	17.3
**Type of employment**	Civil servant	162	89.0
	Partners	14	7.7
	Academia	4	2.2
	Other Private	2	1.1
**Years of experience in public health/epidemiology**	> 5	105	57.7
	3-5	37	20.3
	1-3	32	17.6
	<1	8	4.4

### Role of Rwanda Field Epidemiology and Laboratory Training Program graduates and residents in Marburg virus disease response, Rwanda, 2024

**Fig 1:**
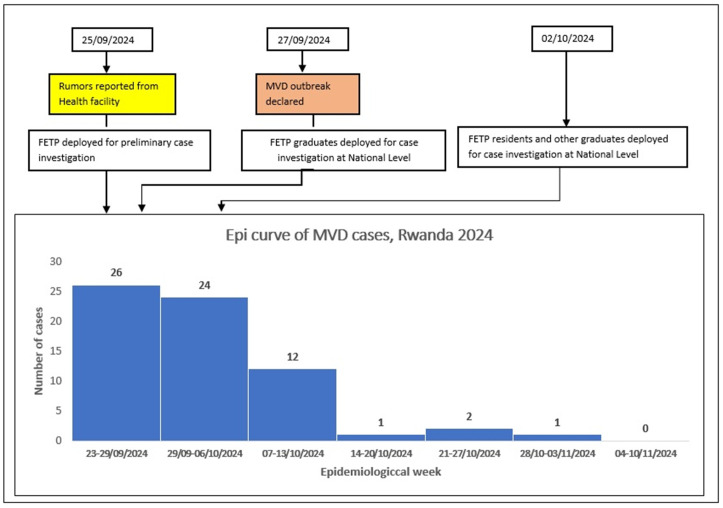
Timeline of Rwanda Field Epidemiology and Laboratory Training Program graduates and resident’s deployment during the Marburg virus disease outbreak in Rwanda, September-November 2024.

An initial group of four RFELTP graduates were deployed as part of a team to conduct a preliminary case investigation on 25 September 2024. The outbreak was declared by the MoH on 27 September 2024. On the same day, ten additional RFELTP graduates, primarily those who worked at the national level (MoH and RBC), and 5 residents were deployed to support the National MVD command post in case investigation and contact tracing. On 2 October 2024, additional graduates and residents from sub-national and private institutions were mobilized to provide support at the national level within the MVD Command Post. All RFELTP advanced tier graduates and residents were first contacted to determine availability. For the residents and graduates who confirmed availability, formal invitation emails were sent to their respective institutions, and all were able to support the response. The RFELTP graduates and residents from the intermediate and frontline tiers who participated in the MVD response supported preparedness and response activities at the health sector levels where they were working (Fig 1.).

**Fig 2:**
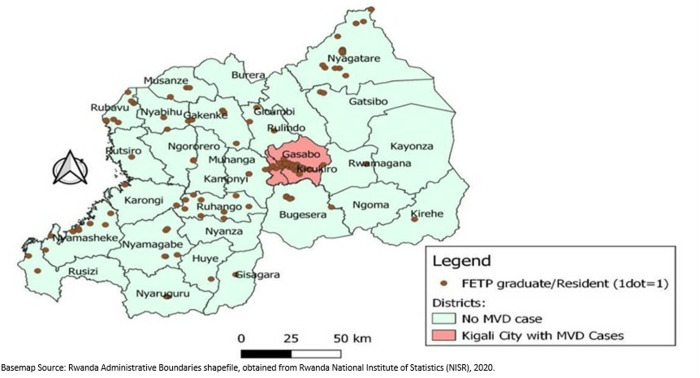
Distribution of Rwanda Field Epidemiology and Laboratory Training Program graduates and residents in Marburg virus disease response (n = 182), by district supported, 2024.

The RFELTP team supported MVD response in all 30 districts of Rwanda; however, the majority were deployed in Kigali City which was the epicenter of the outbreak (Fig 2.).

**Fig 3:**
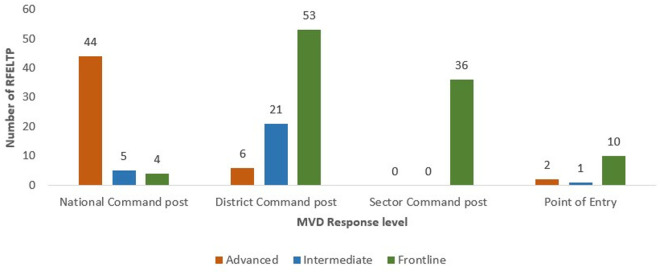
Distribution of Rwanda Field Epidemiology and Laboratory Training Program Tiers by Marburg virus disease response levels, Rwanda, 2024.

The participants involved in the MVD response were from all RFELTP tiers: Advanced, Intermediate, and Frontline. The majority of RFELTP Advanced graduates and residents supported response operations at the national level, while the RFELTP Intermediate and Frontline mainly provided support at the district and sector levels (Fig 3.).

RFELTP graduates and residents were primarily involved in epidemiological surveillance (n = 75, 41.2%), data management (n = 35, 19.2%), infection prevention and control (n = 26, 14.3%) and leadership and coordination (n = 19, 10.4%) (Table 2).

**Table 2:** Rwanda Field Epidemiology and Laboratory Training Program participation in Marburg virus disease response Pillars, September to November 2024, Rwanda.

Pillar	Frequency (n)	Percentage (%)
Epidemiological surveillance	75	41.2
Data management	35	19.2
Infection prevention and control	26	14.3
Leadership and coordination	19	10.4
Laboratory	13	7.1
Risk communication and community engagement	8	4.4
Case management	3	1.7
Vaccination	3	1.7

**Fig 4:**
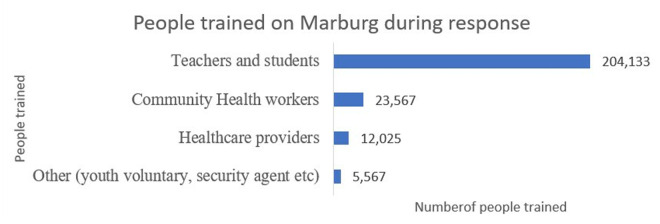
Number of people trained by Rwanda Field Epidemiology and Laboratory Training Program Team on Marburg virus disease preparedness and response, Rwanda, 2024.

RFELTP graduates and residents provided support to District Emergency Operation Centres throughout Rwanda to deliver MVD trainings (Fig 4.). RFELTP graduates and residents visited schools to train teachers, students, and community health workers on MVD signs and symptoms, transmission, preventive measures, and community active case search. Healthcare providers were trained on case investigation forms, donning and doffing of personal protective equipment, hand washing practices, decontamination, waste segregation, final disposal, and other safety precautions. Youth volunteers, a group of young people who contribute to Rwanda’s development without seeking compensation, and other groups of people such as District Administration Security Support Organ staff and the Rwanda National Police staff at the local level were trained to provide support in community sensitization of MVD.

The majority 96.7% of RFELTP graduates and residents felt adequately prepared by their RFELTP training to handle outbreak response. The greatest strengths of their training identified by the REFLTP graduates were in the areas of case investigation, data collection and analysis, and contact tracing. Participants identified advanced analytical data analysis as the most recommended area for further training improvements to improve the role of RFELTP graduates and trainees in future outbreak responses (Table 3).

**Table 3:** Preparedness, Strengths, and Area for improvement by the Rwanda Field Epidemiology and Laboratory Training Program participants, Rwanda, 2024.

Variable	Category	Frequency (n)	Percentage (%)
Feel adequately prepared by REFLTP training to handle the response	Yes	176	96.7
	No	6	3.3
Which aspects of your REFLTP training helped you perform your role during the MVD outbreak response?	Case Investigation	137	20.9
	Data Collection and Analysis	130	19.8
	Contact tracing methods	109	16.6
	Field Safety Protocols	98	14.9
	Coordination and Leadership	96	14.6
	Risk Communication Techniques	87	13.2
What do you think is the biggest strength of REFLTP graduates/trainees in outbreak response?	Case investigation and contact tracing	92	33.4
	Surveillance analysis	74	26.7
	Communication and community engagement skills	54	19.6
	Epidemiological skill	48	17.4
	Laboratory skills	8	2.9
Areas would you recommend for further REFLTP training improvements?	Advanced Data Analysis Skills	93	24.4
	Risk Communication in Outbreak Settings	74	19.4
	Mental Health and Resilience Training	68	17.8
	Rapid Response Field Protocols	62	16.2
	Community Engagement Techniques	52	13.6
	Inter-Agency coordination	33	8.6

## Discussion

This study describes the role of RFELTP graduates and residents during the 2024 Rwanda MVD outbreak. The swift mobilization and deployment of these trained epidemiologists, representing diverse professional backgrounds and geographic locations, underscores the importance of having a well-trained public health workforce. Their comprehensive support primarily focused on key areas such as epidemiology, data management, infection prevention and control, and leadership. The extensive training initiatives facilitated by RFELTP were wide-reaching and essential to the response. Without RFELTP, this considerable group of trained individuals would not have been available to enhance Rwanda’s capacity to respond effectively to this public health emergency.

Survey respondents identified key strengths of RFELTP graduates and residents, such as case investigation, contact tracing, surveillance analysis, and community engagement. These competencies were instrumental in the effective management of the MVD outbreak, particularly in areas such as surveillance and case investigation, where RFELTP teams established a strong reporting mechanism, successfully identified all contacts, tracked the index case, and mapped high-risk groups [18]. These areas of engagement align with the core competencies of the RFELTP, which emphasize data-driven outbreak investigation and management [1,15,19] and are similar to engagement in previous success stories that other Field Epidemiology Training Programs (FETPs) worldwide have demonstrated in fulfilling similar roles during significant outbreaks. For example, FETPs in Liberia proved effective in managing Ebola virus outbreaks through robust case investigation and contact tracing initiatives [20] and in the 2023 MVD outbreak in Tanzania by providing support in surveillance and contact tracing [21].

While most of the advanced tier graduates and residents provided support in Kigali City where all confirmed MVD cases were detected [13], other graduates and residents provided response support in all 30 districts of the country, including at their respective workplaces. This geographical distribution of RFELTP graduates and residents enabled localized interventions that were tailored to the specific needs of communities, including the training of more than 245,000 individuals across the country. The RFELTP team’s role in door-to-door screening and community education aimed to combat inaccuracies in circulating information and build community trust in managing transmission risks. These efforts highlight the benefits of having a three-tiered program that trains individuals at all levels of the health sector to provide support not only at the national level, but in the field and on the ground for enhanced implementation of interventions.

The participants reported high confidence in their RFELTP training to handle the outbreak response with many assuming leadership and coordination roles. RFELTP residents and graduates served as national leads of the surveillance pillar, partner coordination pillar, case investigation team, and contact follow-up team. At the district level, they served as case investigation, infection prevention and control, and laboratory team leads. This high level of reported confidence in their training preparedness and their representation in the response in leadership positions suggests that the program is achieving one of its primary objectives, to effectively build capacity to respond to public health emergencies [1].

Despite the demonstrated strengths of the RFELTP program, participants identified areas for future improvement in the RFELTP curriculum, including advanced data analysis skills, enhanced risk communication in outbreak settings, and mental health and resilience training. These suggestions indicate opportunities for improving the RFELTP curriculum to further strengthen outbreak response capabilities.

This study has several limitations. The use of self-reported data and the provision of phone guidance by the research team may introduce the possibility of response bias and limit the generalizability of the findings. However, the phone support was solely intended to ensure participants fully understood the questions, in order for them to complete the survey. Additionally, the study was conducted within a short time frame of three months, which may not capture the full complexity of the outbreak response.

## Conclusion

Our findings emphasize the essential role of structured field epidemiology training programs like RFELTP in coordinated responses to emerging infectious disease threats. The multidisciplinary approach demonstrated by RFELTP graduates and residents provide a model for building resilient public health systems. The RFELTP has proven to be a valuable resource in outbreak response, producing well-trained, adaptable professionals capable of managing complex public health emergencies. Continued investment and iterative improvements in the RFELTP will not only enhance Rwanda’s public health workforce capacity but will also serve as a model for similar programs in other countries.
